# A Simple Luminescent Adenylate-Cyclase Functional Assay for Evaluation of *Bacillus anthracis* Edema Factor Activity

**DOI:** 10.3390/toxins8080243

**Published:** 2016-08-18

**Authors:** Ma’ayan Israeli, Shahar Rotem, Uri Elia, Erez Bar-Haim, Ofer Cohen, Theodor Chitlaru

**Affiliations:** Department of Biochemistry and Molecular Genetics, Israel Institute for Biological Research, Ness-Ziona 74100, Israel; maayani@iibr.gov.il (M.I.); shaharr@iibr.gov.il (S.R.); urie@iibr.gov.il (U.E.); erezb@iibr.gov.il (E.B.-H.); oferc@iibr.gov.il (O.C.)

**Keywords:** *Bacillus anthracis*, Edema Factor, adenylate cyclase, luminescent assay, luciferase, ATP-depletion

## Abstract

Edema Factor (EF), the toxic sub-unit of the *Bacillus anthracis* Edema Toxin (ET) is a calmodulin-dependent adenylate cyclase whose detrimental activity in the infected host results in severe edema. EF is therefore a major virulence factor of *B. anthracis*. We describe a simple, rapid and reliable functional adenylate-cyclase assay based on inhibition of a luciferase-mediated luminescence reaction. The assay exploits the efficient adenylate cyclase-mediated depletion of adenosine tri-phosphate (ATP), and the strict dependence on ATP of the light-emitting luciferase-catalyzed luciferin-conversion to oxyluciferin, which can be easily visualized. The assay exhibits a robust EF-dose response decrease in luminescence, which may be specifically reverted by anti-EF antibodies. The application of the assay is exemplified in: (a) determining the presence of EF in *B. anthracis* cultures, or its absence in cultures of EF-defective strains; (b) evaluating the anti-EF humoral response in experimental animals infected/vaccinated with *B. anthracis*; and (c) rapid discrimination between EF producing and non-producing bacterial colonies. Furthermore, the assay may be amenable with high-throughput screening for EF inhibitory molecules.

## 1. Introduction

The gram-positive spore-forming bacterium *Bacillus anthracis* is the causative agent of anthrax, a rare fatal disease that is initiated, in its most severe form, by inhalation of infectious spores. Due to the severity of the disease, the respiratory infection and the everlasting resistance of the spores to unfavorable environmental conditions, *B. anthracis* is considered a potential biological warfare agent [1,2,3,4,5] and included in the Center for Disease Control (CDC) list of select agents.

*B. anthracis* produces two exotoxins, Lethal Toxin (LT) and Edema Toxin (ET). ET and LT, together with the poly d-glutamic acid capsule, are considered to represent the three “classic” virulence factors of the bacteria, necessary for the manifestation of the disease in a wide variety of hosts [6,7,8,9,10]. ET and LT are binary toxins that include, in addition to their specific sub-unit effectors EF (Edema Factor) and LF (Lethal Factor), respectively, the common subunit PA (Protective Antigen). The genes *pag*, *lef* and *cya*, encoding for PA, LF and EF, respectively, are located on the native plasmid pXO1, harbored by all virulent *B. anthracis* strains. Functions required for the biosynthesis of the poly d-glutamic acid capsule, are encoded by genes located on the pXO2 native plasmid harbored by *B. anthracis*. PA is not toxic by itself, yet it fulfills the central role of recognizing receptors on the membrane of host cells and translocating the toxic subunits EF and LF into the cytoplasm of the host cells [8,11,12]. EF is a Ca^2+^ and calmodulin-dependent adenylate cyclase (AC) which is essential to the pathogenesis of the bacteria [13]. EF increases the concentration of cyclic adenosine monophosphate (cAMP) in the cells (by 1000-fold by converting 20%–50% of the available ATP) resulting in a decrease in chloride ions and increased water efflux from affected cells, which leads to massive edema observed in cutaneous cases of anthrax. EF also inhibits the phagocytic and oxidative burst activity of neutrophils, which increases the vulnerability of the host to widespread infection [10,11,14,15,16]. EF was reported to affect phagocyte function, phagocytic abilities of monocytes, cytokine secretion of dendritic cells due to the extreme elevation of the cAMP concentration above physiological levels (see [13] for review of the detrimental consequences of EF activity).

The study of *B. anthracis* pathogenesis, anthrax disease progression in general, and the role of the bacterial toxins in particular, depends on the availability of experimental assays which enable detection, quantification and assessment of the physiological enzymatic activity of EF and LF. As of today, the enzymatic activity of EF is determined by measuring the concentration of the cAMP resulting from the EF-mediated conversion of ATP, either by radioactive labeling with [α-^32^P]-ATP and subsequent chromatographic fractionation for separation of the radioactive cAMP product [11,17,18] or by the use of specific anti-cAMP antibodies [19,20,21].

Owing to the simplicity of light emission measurement, luciferase/luciferin reaction is widely used in various functional and/or visualization biological assays. Likewise, the luciferase gene is one of the most frequently employed reporter genes in studies addressing protein expression in a quantitative manner. The light emitting reaction mediated by luciferase is highly sensitive, exhibits in most of the cases a superior signal-to-noise ratio and a wide dynamic range (for reviews on the extensive experimental implementations of luciferase-mediated luminescence, see [22,23,24]). Furthermore, the quantitative measurement of the light employs relatively simple devices and does not require sophisticated expensive equipment.

In this report, we document a novel simple, rapid and sensitive functional assay for EF activity based on the ability of this enzyme to promote efficient depletion of ATP which may be quantitatively evaluated by inhibition of a luciferase-mediated light-emitting reaction. We show that this simple and cost-effective method may be implemented for evaluation of EF activity in crude *B. anthracis* culture supernatants, may be used for detection of anti-EF antibodies or other EF-inhibitory molecules. Moreover, this method is amenable with high-throughput screens.

## 2. Results and Discussion

### 2.1. An ATP-Depletion Luminescence-Assay for Detection and Evaluation of the AC Activity of EF

**Figure 1:**
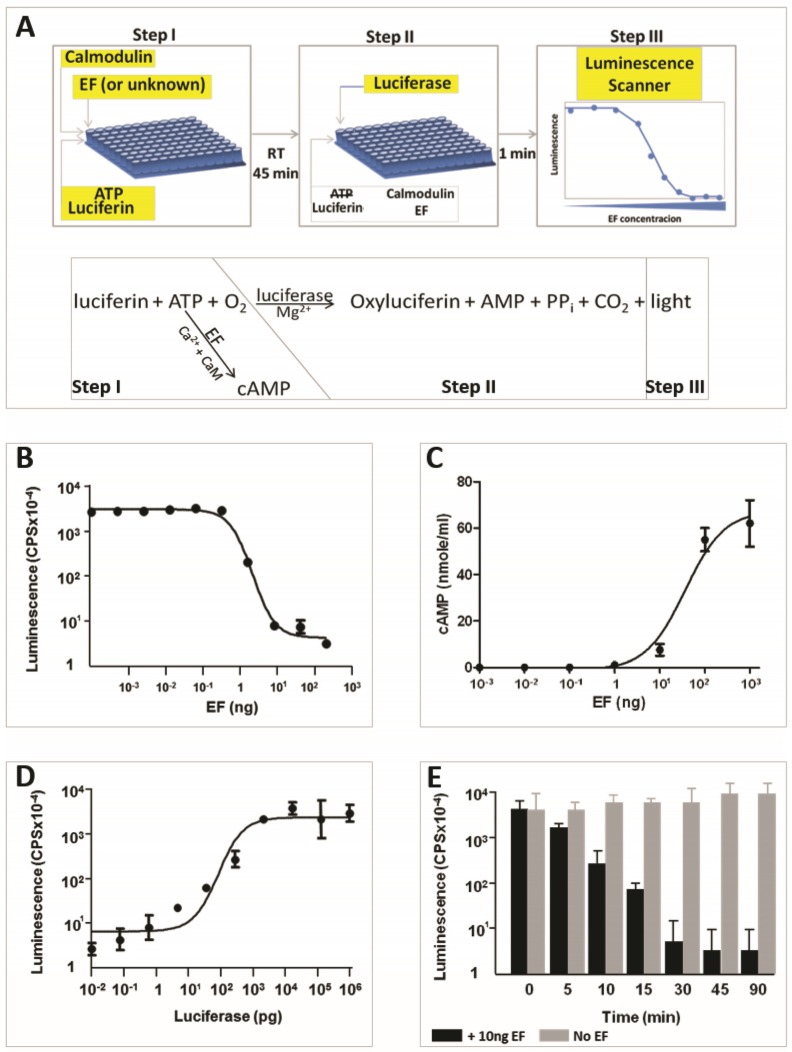
A luminescent adenosine tri-phosphate (ATP)-depletion assay for identification of edema factor (EF), based on its adenylate cyclase activity. (**A**) Schematic description of the assay. Step I: EF or “unknown” samples are incubated together with Calmodulin (CaM), ATP and luciferin in microtiter plate wells for the indicated period of time at room temperature. Step II: Luciferase is added to the wells. Step III: The microtiter plate is scanned in a luminescence scanner. The amount of luminescence is directly proportional to the amount of ATP in the reaction which decreases due to the AC activity of EF. The light emitting reaction is described as an equation under the scheme. (**B**) Dose-response curve of luminescence as a function of the amount of EF in the reaction. (**C**) The dose-dependent decrease in luminescence caused by EF is paralleled by an increase in the amount of cyclic adenosine mono-phosphate (cAMP) resulting from the conversion of ATP. (**D**) Dose-response of the luminescence as a function of the amount of luciferase added at step II of the assay. Routinely, an amount of luciferase affording maximal light emission (100 ng) was used in the assay. (**E**) Time course of the luminescence decrease in the presence of 10 ng EF (black histograms) compared to luminescence in the absence of EF (gray histograms). In panels **B**–**E**, luminescence is expressed in photon *counts per second* (cps); this value depends on the sensitivity and the internal calibration of the scanner and may vary in different scanners. Reactions in the experiments described in **B**–**D** were carried out for 3 h. The data in panels **B**–**E** represent the geometrical mean (±SD) obtained in at least three independent experiments. See Experimental Section for details.

We reasoned that due to its strict dependence on ATP (see also Supplementary Materials Figure S1), the luciferase-luciferin reaction may represent a sensitive means to determine ATP availability and consequently, the presence and function of enzymes which catalyze ATP formation or depletion. Accordingly, the reaction may be exploited for determining presence of functional EF whose AC activity results in depletion of ATP. Indeed, when the luminescence reaction buffer (containing luciferin and ATP) was pre-incubated for 2 h with increasing amounts of purified EF (see Experimental Section and schematic depiction of the assay in Figure 1A) prior to the addition of luciferase, a net dose-dependent decrease in the level of emitted light was observed (Figure 1B). The data in Figure 1B showed that the luminescence-inhibitory effect of EF could be determined along a wide dynamic range of the photo-signal (>3 orders of magnitude) and exhibited a very high signal-to-noise ratio. A decrease of at least one order of magnitude in luminescence was consistently recorded following pre-incubation with as little as 1 ng EF and reached a maximal level at 10 ng EF. Of note, the effect of pre-incubation with EF was dependent on the presence of the cofactor calmodulin, in line with the known requirement of this cofactor for manifestation of its AC activity [16,25].

To confirm that the EF-mediated decrease in luminescence reflected the conversion of ATP to cAMP, the cAMP concentration in the reaction mixture following incubation with increasing amounts of EF (Step 1 in Figure 1A) was determined by quantitative ELISA. We observed that the decrease in luminescence was clearly proportional to the synthesis of cAMP from ATP (Figure 1C). The amount of luciferase used in the assay (200 ng) was determined to afford maximal light emission (Figure 1D). While the ATP-depletion experiment depicted in Figure 1B was performed by pre-incubating the luciferin reagent (containing ATP) with EF for 3 h, we noted that significant inhibition of the reaction was observed within 10–15 min and reached a maximal level following 45 min of incubation (Figure 1E). Prolonging the incubation with EF up to 24 h did not result in enhancement of the sensitivity of the assay.

### 2.2. Implementation of the EF-Mediated ATP-Depletion Luminescence-Assay for Evaluation of Active EF in Bacillus anthracis Cultures

**Figure 2:**
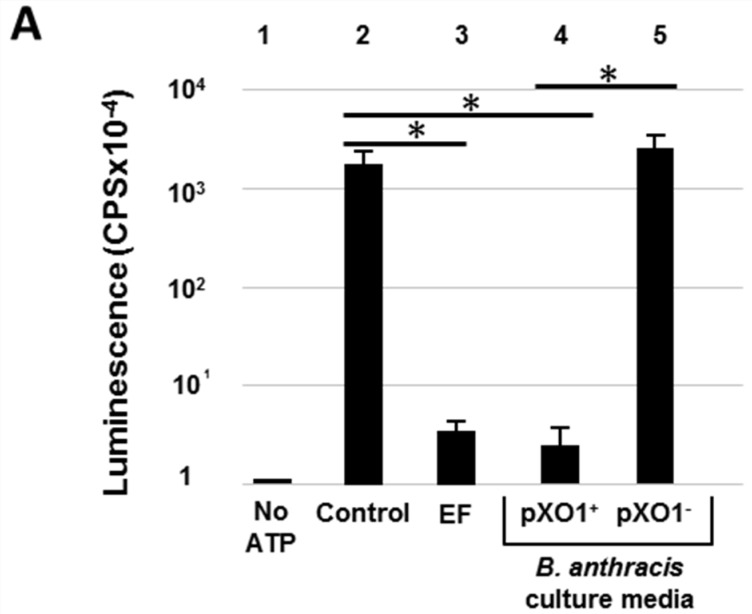

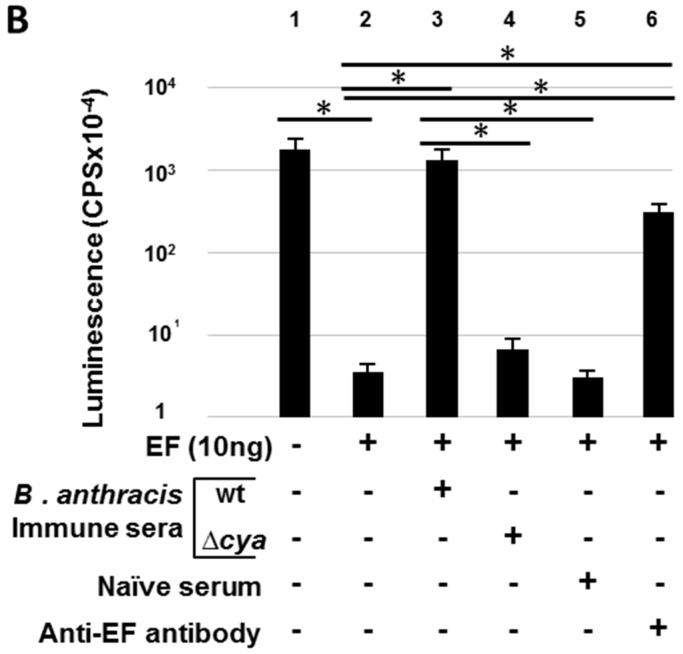
The luminescent ATP-depletion assay is implemented for determining the presence of active EF in *B. anthracis* culture supernatants and the neutralizing activity of anti-EF antibodies. (**A**) Presence of EF in *B. anthracis* culture-supernatants. Histogram 1: Luminescence in the absence of ATP. Histogram 2 (control): Luminescence measured in the luciferase/luciferin reaction. Histogram 3: Luminescence obtained following addition of 10 ng pure EF. Histograms 4–5: Luminescence following addition of 10 μL of *B. anthracis* cultures (8 h growth in DMEM [Dulbecco’s modified Eagle’s medium] in an 8% CO_2_-enriched atmosphere at 37 °C) of WT (wild-type) Vollum (pXO1^+^, histogram 4) or ∆Vollum (pXO1^−^, histogram 5) strains. (**B**) Inhibitory effect of EF is reversed by anti-EF antibodies. Histogram 1 (control): Luminescence measured in the luciferase/luciferin reaction. Histogram 2: Luminescence inhibition promoted by EF. Histogram 3–5: Effect of inclusion of sera. Reversion of EF inhibitory effect is promoted by pre-incubation of EF with sera obtained from guinea-pigs immunized with an EF producing *B. anthracis* strain (histogram 3) but not with sera from animals immunized with a *B. anthracis* ∆*cya* strain (histogram 4) or by sera from naïve animals (histogram 5). Histogram 6: EF-dependent luminescence inhibition is reversed by pre-incubation of EF with commercial anti-EF antibodies (10 μL) in the reaction. Luminescence is expressed in photon counts per second (cps). The experiment was independently performed at least three times in triplicate. The data represent the average luminescence level (+SD) obtained in a representative experiment. * Statistical analysis of the inhibitory effect of EF on luminescence was performed using the student *t*-test (*p* < 0.001). The presence (+) or absence (-) of a particular element included in the assay is indicated in the table below the histogram chart.

The ATP-depletion assay described above was validated in determining the presence of EF-associated AC activity in the supernatant of a *B. anthracis* cultures (Figure 2). Secretion of EF, LF and PA is induced by culturing *B. anthracis* in a CO_2_ elevated atmosphere, conditions which are considered to mimic those encountered by the bacteria in the host [26,27,28]. Indeed, supernatants collected from CO_2_-induced cultures of a *B. anthracis* pXO1-exhibiting strain were highly efficient in inhibiting the luciferase-mediated luminescence similar to the inhibitory effect of purified EF (Figure 2A, compare histogram 2 to histograms 3 and 4) while similar supernatants collected from a strain cured of the pXO1 plasmid (and therefore devoid of EF) failed to induce any decrease in the level of luminescence (Figure 2A, histogram 5). Obviously, the EF-mediated ATP-depletion functional assay is inherently limited to the detection of active EF; yet the assay could be employed for quantitatively determining the amount of active EF in culture samples, by comparing the luminescence emitted by serial dilutions of an “unknown” sample with an EF calibration curve (similar to the EF dose-response curve in Figure 1B). Such a quantitative determination of EF is exemplified in Supplementary Materials Figure S2. Furthermore, the assay was employed to determine the kinetics of secretion/accumulation of active EF in the supernatant of the *B. anthracis* culture (Supplementary Materials Figure S3).

The assay could be further implemented in experiments that addressed the efficiency of EF production in a variety of media. Consequently, it is routinely employed for optimization of culturing conditions favorable for maximizing EF synthesis. Furthermore, the assay serves for evaluating loss of function of EF or for confirming the absence of EF from cultures or culture-fractions in which the presence of EF is not desired.

### 2.3. Specific Reversion of EF Activity by Anti-EF Antibodies

AC activity is associated not only with *B. anthracis* EF but also with several secreted virulence factors of other pathogenic bacteria [16,25]. Therefore, theoretically, the ATP-depletion assay could detect AC activity, which does not necessarily reflect presence of EF. We therefore reasoned that modification of the assay by inclusion of specific antibodies would establish that the detected AC activity could be attributed to EF. Indeed, we observed that a 45 min pre-incubation of specific anti-EF antibodies with EF prior to the EF-ATP incubation step (Step 1, Figure 1A), significantly reverted the inhibitory effect of EF on the luminescence reaction resulting in an increase of more than two orders of magnitude in luminescence (Figure 2B, compare histograms 2 and 6). Accordingly, the ability of anti-EF antibodies to revert the inhibitory effect of EF in the ATP-depletion assay could be implemented for determining the presence of anti-EF antibodies in plasma samples. Indeed, the data depicted in Figure 2C showed that while full reversion of the EF inhibitory effect occurred upon incubation with anti-sera collected from the circulation of guinea pigs vaccinated with an EF-producing *B. anthracis* Sterne strain (Figure 2B, compare histograms 2 and 3), no effect was promoted by sera collected from animals immunized with a *B. anthracis* ∆*cya* strain or from non-infected naïve animals (histograms 4 and 5). Thus, the data demonstrate that the luminescence-inhibition assay can be readily used for evaluation of the humoral anti EF response elicited in animals infected or vaccinated with *B. anthracis*. Of note, since the test is based on the AC activity of EF, evaluation of the anti-EF humoral response using the luminescence-depletion assay detects only EF-neutralizing antibodies which probably represent a sub-population of the total anti-EF antibodies elicited by vaccination.

### 2.4. Implementation of the ATP-Depletion Assay for Detection of EF-Mutated Bacteria

**Figure 3:**
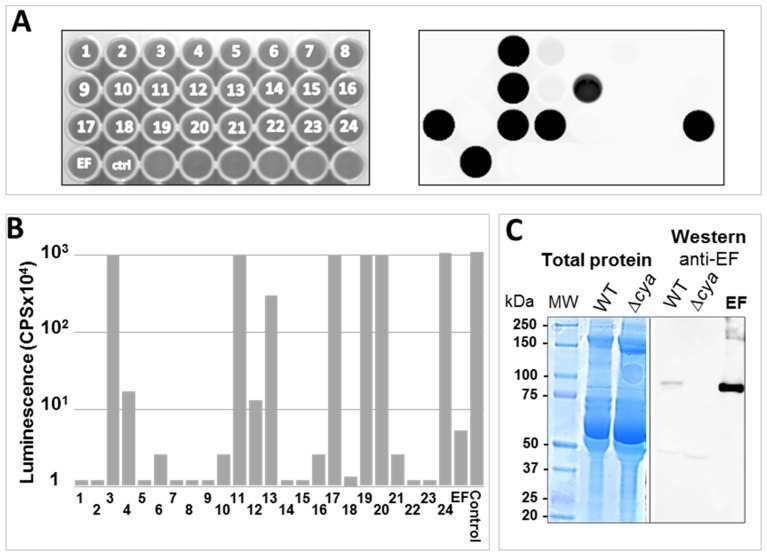
The luminescent ATP-depletion assay is implemented to distinguish between wild-type (WT, parental) and *cya*-gene disrupted colonies of *B. anthracis*. (**A**) 24 discrete colonies obtained after gene disruption of the *cya*-gene by a homologous recombination gene-targeting protocol [29,30,31] were used to inoculate 24 wells of a micro-titer plate (left panel) containing 200 μL DMEM and grown for 2 h. Ten microliters of the supernatant of each well were transferred to an ATP-depletion luminescence-inhibition assay microtiter plate (as described in Figure 1A). The assay was carried-out for 45 min. Following Luciferase addition, the plate was analyzed for 10 s with a luminescent-image analyzer (right panel). Luminescent wells (visualized as black dots) indicate that the bacteria did not secrete functional EF: (**B**) quantitative analysis of the plate by the VICTOR^3^TM luminometer; and (**C**) confirmation of abrogation of EF synthesis by Western-blot analysis of colony 3 (which did not exhibit luminescence inhibition in the assay, marked ∆*cya*) compared to colony 5 (which inhibited the luminescence in the ATP-depletion assay, marked WT-wild type); all EF-devoid colonies selected by the luminescent assay demonstrated the desired phenotype by Western-blot analysis.

Considering the simplicity of evaluating the presence of EF in bacterial cultures, we attempted to use the luminescence assay for discerning between *B. anthracis* colonies exhibiting deletions in the *cya* gene (which abrogate the synthesis of EF) and WT colonies. Indeed, in the course of generating a ∆*cya* strain (by targeted gene disruption), a screening procedure for identifying colonies exhibiting the desired genetic manipulation (full deletion of the *cya* gene) amongst WT colonies, was carried out using a simplified multi-titer plate-adjusted ATP-depletion luminescence assay. Figure 3 summarizes a screening procedure in which individual wells in a multi-titer plate (containing DMEM media) were inoculated with individual bacterial colonies (by means of a microbiological loop) and incubated for 2 h in a CO_2_-enriched atmosphere. A small sample of the culture supernatant (10 μL) was then transferred from all individual wells to a twin micro-titer plate containing the ATP/luciferin/calmodulin buffer (Step 1 in Figure 1A). Following 45 min of incubation, luciferase was added as described above (Figure 1A). The plate was exposed for 10 s directly on a luminescence-reader analyzer for immediate evaluation of luminescence (Figure 3A, right panel) or quantitatively analyzed on a luminescence-counter (Figure 3B). By both procedures, colonies failing to inhibit the luminescence reaction could be readily identified suggesting that these colonies indeed exhibited the desired deletion mutation, which abrogated the synthesis of EF. The desired phenotype was further confirmed by Western blot analysis (Figure 3C) and the correct genetic manipulation was established by PCR analysis and chromosomal-DNA sequencing of the target genomic locus (Supplementary Materials Figure S4). Taken together, the data indicate that the ATP-depletion luminescence assay may be adapted easily for a high-throughput procedure, which rapidly probes the EF activity.

In summary, the data documented in this report demonstrate that a simple ATP-depletion assay can serve as a valuable tool for evaluating the AC activity of EF and implemented for quantitatively determining the presence of functional EF. The assay can detect as little as 1 ng EF (Figure 1B), a sensitivity level which is compatible with detection of EF in a variety of bacterial culture conditions. Indeed, using this assay EF could be detected in *B. anthracis* cultures and could be implemented to distinguish between EF producing and non-producing *B. anthracis* strains (Figure 2 and Figure 3). Of note, a concentration of 2–6 μg/mL EF was reported in the circulation of infected rabbits 48–72 h post-infection [32]. Accordingly, the ATP-depletion assay, which exhibits a sensitivity level of 0.1 μg/mL, may be theoretically implemented for detection of EF in infected animals.

The importance of the assay resides in its simplicity and rapidity. The assay is homogenous, in the sense that all ingredients are present in the reaction mixture (Step 1, Figure 1A) except the enzyme luciferase which is introduced in the same reaction tube 15–45 min later (Step 2, Figure 1A). It does not require any purification step prior to the reading step (step 3); this is not the case with the AC assays based on radioactive determination of the cAMP concentration, which require a chromatography fractionation final step for purification of the product, nor does it require the use of radioactive material. The EF level of detection exhibited by the assay is not inferior to that of a commercial anti-cAMP ELISA (or even slightly superior, Figure 1B,C). We note that the assay described here is less sensitive than a radioactive ATP conversion assay which exhibits a detection level of 10–50 ng/mL EF (see for example [11]), yet it is considerably simpler than both of the alternative assays. Luminescence is a physical phenomenon which can be easily measured with readily available non-sophisticated scanners or any device which quantifies photon emission such as scintillation analyzers, β-counters or luminescent image-analyzers. Since 15 min is often sufficient to achieve significant depletion of ATP (Figure 1E), the assay may be carried-out within less than 30 min. Theoretically, the assay may be implemented for detection of other virulence factors which carry AC activity, such as the CyaA factor of *Bordetella pertussis* [25,33,34,35] or the exotoxin ExoY of *Pseudomonas aeruginosa* [36]. Indeed we used the assay successfully for measuring the activity of a commercial preparation of *B. pertusis* CyaA. Of note, *P aeruginosa* ExoY AC activity does not require calmodulin as a cofactor [16,36], therefore calmodulin-dependence can be used as a parameter for distinction between ExoY and CyaA or EF. Here we show that by using anti EF antibodies, the AC activity is specifically blocked preventing ATP depletion, and consequently establishing that the AC activity measured in the assay can be attributed to EF (Figure 2B). Therefore, inclusion of anti-EF antibodies may confer to the assay a high level of specificity. Further to this observation, the sensitivity of the assay to anti-EF antibodies enables its implementation for determining the presence of specific antibodies. Indeed, a specific anti-EF humoral response could be determined in animals immunized with an EF-producing *B. anthracis* strain (but not in control animals vaccinated with EF non-producing *B. anthracis* bacteria, Figure 2B) by assaying the ability of serum samples to revert the EF-mediated luminescence inhibition.

Finally, the ATP-depletion luminescence assay rapidly and accurately distinguished between *B. anthracis* EF-producing colonies and ∆*cya* colonies in which EF synthesis was abrogated by targeted disruption of the *cya* gene (Figure 3). This observation strongly suggests that the assay may be applied for high-throughput screening, not only for determining the presence of EF in a given sample, but also for identification of specific inhibitors of its AC activity. Such studies, aimed at the identification of potential new specific anti-EF inhibitory molecule exhibiting therapeutic potential, are currently being conducted.

## 3. Experimental Section

### 3.1. Reagents

The recombinant rEF (HTE1/1) used in this study is a His-tagged version of EF carrying 6 His residues at its *C*-terminus produced by a Vollum strain of *B. anthracis* generated by Haim Levy from IIBR. EF purified by Zn-affinity chromatography was kindly provided by the Department of Biotechnology at IIBR. The purified preparation of EF was quantified by ELISA and analyzed by SDS-gel electrophoresis which evidenced >95% purity. Rabbit anti-EF antibodies used for specific reversion of luminescence inhibition, were purchased from Thermo Scientific (Pierce PA5-20095, Rockford, IL, USA). The anti-EF antibodies used for Western-blot analysis were previously described [37,38]. Immune anti-*B. anthracis* serum used in the experiment described in Figure 2B was collected from guinea-pigs vaccinated with *Sterne* ∆*htrA* attenuated *B. anthracis* strain [37] or with a *Sterne* ∆*htrA* ∆*cya* (see below). Sera were collected and pooled from 5 animals, 5 weeks post-vaccination with 2 doses of 10^9^ spores (5 weeks apart). All studies involving experimental animals were carried out according to the National Research Council Guide for the Care and Use of Laboratory Animals and approved by the IIBR Animal Use Committee (Protocol GP-16-2012, approved on 29 October 2012). 

### 3.2. Bacterial Strains

Four attenuated *B. anthracis* strains were used in this study. *Sterne* (pXO1^+^; pXO2^−^), *Sterne* ∆*htrA* (*htrA* disrupted strain devoid of antibiotic resistance, designated in the IIBR collection BA106 [37]), and ∆*Vollum* (pXO1^−^; pXO2^−^; non-toxinogenic and non-encapsulated strain [38]) and *Sterne* ∆*htrA* ∆*cya* (cya-gene disrupted strain devoid of antibiotic resistance, IIBR collection BA110, [39]). All *B. anthracis* strains used exhibited indistinguishable growth profiles in all culturing media. The *B. anthracis* ∆*cya* strain described in Figure 3 was generated by a markerless gene replacement method [29] modified as reported previously [30,31].

### 3.3. Cultivation and Sampling

Strains were cultivated as described previously [38]. In brief, clonal cells were inoculated into a tube with 3 mL Luria-Bertani (LB) liquid medium at 37 °C with vigorous agitation for 6 h. From this culture, 200 µL were used to inoculate another tube with 15 mL DMEM (Biological industries, Beit Haemek, Israel) supplemented with 10% FCS (Biological industries, Beit Haemek, Israel). The tube was incubated overnight at 37 °C in a 10% CO_2_ atmosphere. The culture supernatant was collected by centrifugation (5 min; 4000 × RPM) and was filtered twice through a 0.22-µm filter. All samples were stored frozen (−20 °C) until analysis.

### 3.4. Adenylate Cyclase Assay

The novel AC assay is described in Figure 1A; 10 µL of sample (purified EF or culture supernatant) were added to 50 µL of luciferase assay reagent (Promega E1483, Madison, WI, USA) containing 1 µM Calmodulin (Sigma, C4874, St-Louis, MO, USA) and 0.5 mM CaCl_2_ (Sigma, TA374481, St-Louis, MO, USA) in a polysorp fluoroNunc microtiter plate (Nunc, Roskilde, Denmark) and incubated for at least 45 min (or for the periods of time indicated in the various experiments) at 30 °C. Ten microliters of 1.437 µg/mL QuantiLum^®^ Recombinant Luciferase (Promega E1701, Madison, WI, USA) were added to the mixture and luminescence was measured immediately with a luminometer (VICTOR^3^TM, Perkin Elmer, Waltham, MA, USA). In the case of EF inhibition by antibodies, 10 µL (1 μg/μL) of EF were incubated for 45 min at room temperature with 5 µL of antibodies (Rabbit anti-EF, Pierce, Thermo-scientific, Rockford, IL, USA) or anti-sera (see below); 10 µL from this mixture were then added to the luciferase assay reagent and the assay was continued as described. In the experiment describing identification of *cya*-gene disrupted colonies (Figure 3), the microtiter plate was analyzed both by exposure to a Fujifilm LAS-3000 luminescent image-analyzer (Fuji, Tokyo, Japan), (Figure 3A) and then scanned quantitatively with the VICTOR^3^TM luminometer (Figure 3B). The strict dependence of the luciferase-mediated reaction on ATP (Supplementary Materials Figure S1) was determined by performing the luciferin/luciferase reaction in the presence of increasing concentrations of ATP. In all experiments, luminescence is measured by CPS (counts per seconds), the arbitrary units describing photon detection by the VICTOR^3^TM luminometer. These values depend on the internal calibration of the luminometer.

### 3.5. Western Blot Analysis

Electrophoresis of supernatant samples was performed using 4%–12% SDS-PAGE (NuPage Bis-Tris, Invitrogen, Carlsbad, CA, USA), with 10 µL of culture supernatant loaded for each sample, using Precision Plus Molecular weight markers (Bio-Rad, Hercules, CA, USA). Western blots were generated using the Nitrocellulose Western iBlot Gel transfer Semi-dry system (Invitrogen, Carlsbad, CA, USA). Visualization of immunoreactive bands was carried out by an ECL (electro-chemo-luminescence) reaction (Pierce Supersignal West Pico Chemiluminescent substrate kit, Thermo Scientific, Rockford, IL, USA) mediated by peroxidase-conjugated secondary antibodies (Amersham) and detected by the FUJIFILM LAS-3000 detection system (Fuji, Tokyo, Japan). For this, 1:500 diluted primary antibody rabbit anti-EF and 1:4000 diluted secondary antibody donkey anti-rabbit were used.

### 3.6. cAMP Quantification by ELISA

A commercial cAMP ELISA kit (Cell Biolabs, San Diego, CA, USA) was used to determine total cAMP concentration following the manufacturer’s instructions.

### 3.7. Data Analysis

EF-dependent luminescence inhibition curves and histograms were computer-generated and analyzed using the GraphPadPrism (Version 5, San Diego, CA, USA, 2007) statistical analysis software. The equation describing the dose-dependent effect of EF concentration on luminescence (Figure 1B–D and Supplementary Materials Figure S2) was determined by the non-linear regression of the log values. A *t*-test was used to compare the mean luminescence inhibition between experimental groups. A *p*-value of <0.05 was considered statistically significant.

## Supplementary Materials

The following are available online at www.mdpi.com/2072-6651/8/8/243/s1. Figure S1: Strict dependence of luciferase-catalyzed luminescence emission on the ATP concentration. The chemical conversion of luciferin into oxyluciferin mediated by luciferase is described in the upper panel. Figure S2: Quantitative determination of the EF concentration of a sample using an EF-dose dependence luminescence-inhibition calibration curve. A calibration-curve (black dots) was set up with serial dilutions of a pure-EF preparation and the ATP-depletion luminescence-inhibition assay was performed as described (see Figure 1A and Experimental Section). The dotted black line indicates the ED50 (Effective Dose 50%) of the EF concentration, at which the EF-dependent inhibition is 50% of the maximal observed inhibition. In parallel, the assay was performed with serial dilutions of an “unknown” EF-containing sample of a *B. anthracis* filtered culture supernatant. The equation describing the dose-dependent effect of EF concentration on luminescence was determined by the non-linear regression of the log values (Experimental Section); the concentration of EF in the sample was determined to be 0.8 g/mL by the ATP-depletion assay. Figure S3: Accumulation of AC activity in *B. anthracis* culture supernatant. The presence of EF in samples collected from a *B. anthracis* culture (in DMEM in 10% CO2 atmosphere) was determined by the ATP-depletion assay at the indicated time-points. The luminescence-inhibition is indicated by the blue histograms (right axis in blue). The growth profile of the culture is indicated by the black curve (left axis in black) super-imposed on the luminescence-inhibition determined by the ATP-depletion assay. Figure S4: Confirmation of the *cya*-gene disruption by PCR analysis. PCR reactions were carried-out using as templates chromosomal DNA prepared from Colony No. 3 (which exhibited abrogation of the AC activity see Figure 3) and parental wild-type (WT) strain. The deletion of the *cya* gene is schematically depicted in the upper panel as a dotted line. UTR refers to the homology fragments flanking the *cya* locus which were used for the targeted disruption. The position of the primers used for the PCR reactions is indicated as arrows. The expected length (in base pairs) of the PCR products as well as the DNA-sequence of the primers are indicated in the table in the right-lower panel. The PCR products were further analyzed by DNA sequencing which confirmed the deletion of the *cya* gene.
