# Supplementary material for: A Simple Luminescent Adenylate-Cyclase Functional Assay for Evaluation of Bacillus anthracis Edema Factor Activity
Source: Toxins (Basel). 2016 Aug 18;8(8):243. doi: 10.3390/toxins8080243 (PMC4999859; doi:10.3390/toxins8080243)
Supplement: Supplementary file 1 [file toxins-08-00243-s001.pdf]

Supplementary Materials: A Simple Luminescent Adenylate-Cyclase Functional Assay for Evaluation of *Bacillus anthracis* Edema Factor Activity

Ma'ayan Israeli, Shahar Rotem, Uri Elia, Erez Bar-Haim, Ofer Cohen and Theodor Chitlaru

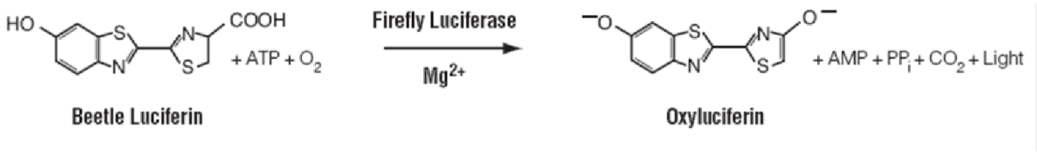

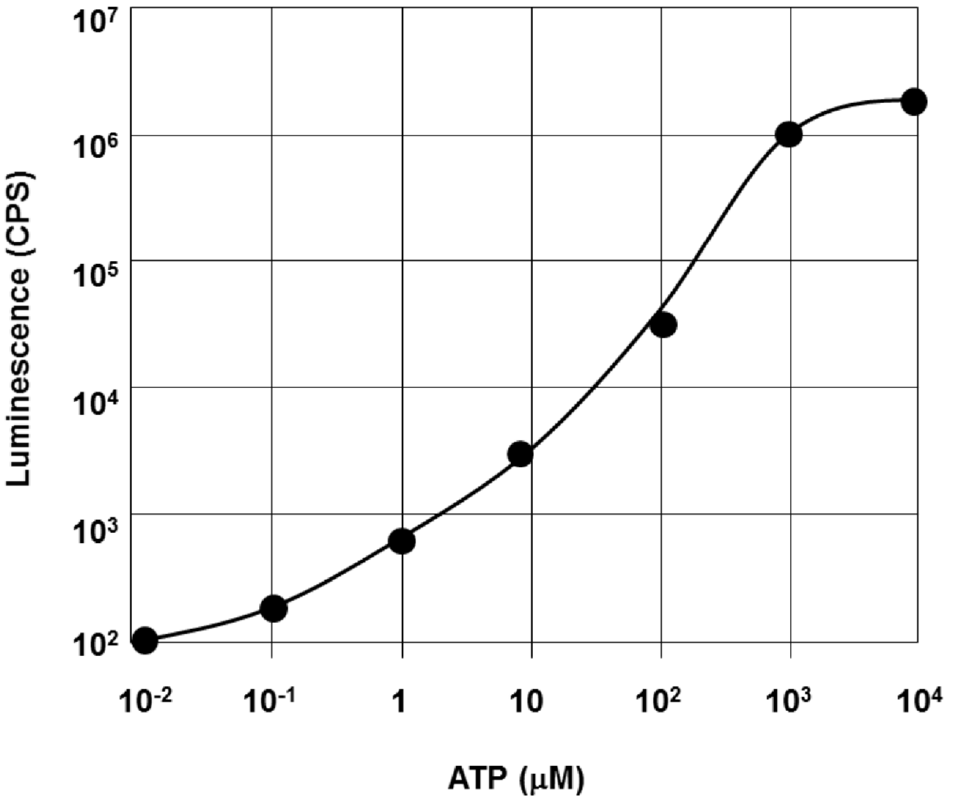

Figure S1. Strict dependence of luciferase-catalyzed luminescence emission on the adenosine triphosphate (ATP) concentration. The chemical conversion of luciferin into oxyluciferin mediated by luciferase is described in the upper panel.

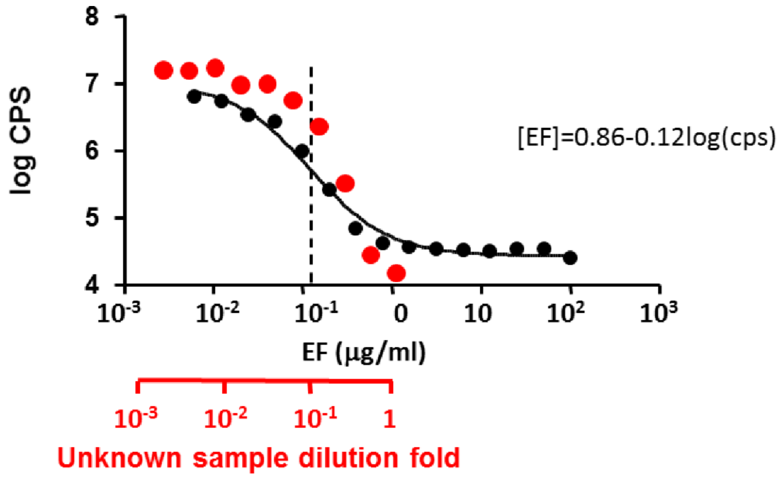

Figure S2. Quantitative determination of the Edema factor (EF) concentration of a sample using an EF-dose dependence luminescence-inhibition calibration curve. A calibration-curve (black dots) was set up with serial dilutions of a pure-EF preparation and the ATP-depletion luminescence-inhibition assay was performed as described (see Figure 1A and Experimental Section). The dotted black line indicates the ED50 (Effective Dose 50%) of the EF concentration, at which the EF-dependent inhibition is 50% of the maximal observed inhibition. In parallel, the assay was performed with serial dilutions of an "unknown" EF-containing sample of a *B. anthracis* filtered culture supernatant. The equation describing the dose-dependent effect of EF concentration on luminescence was determined by the non-linear regression of the log values (Experimental Section); the concentration of EF in the sample was determined to be 0.8 μg/mL by the ATP-depletion assay.

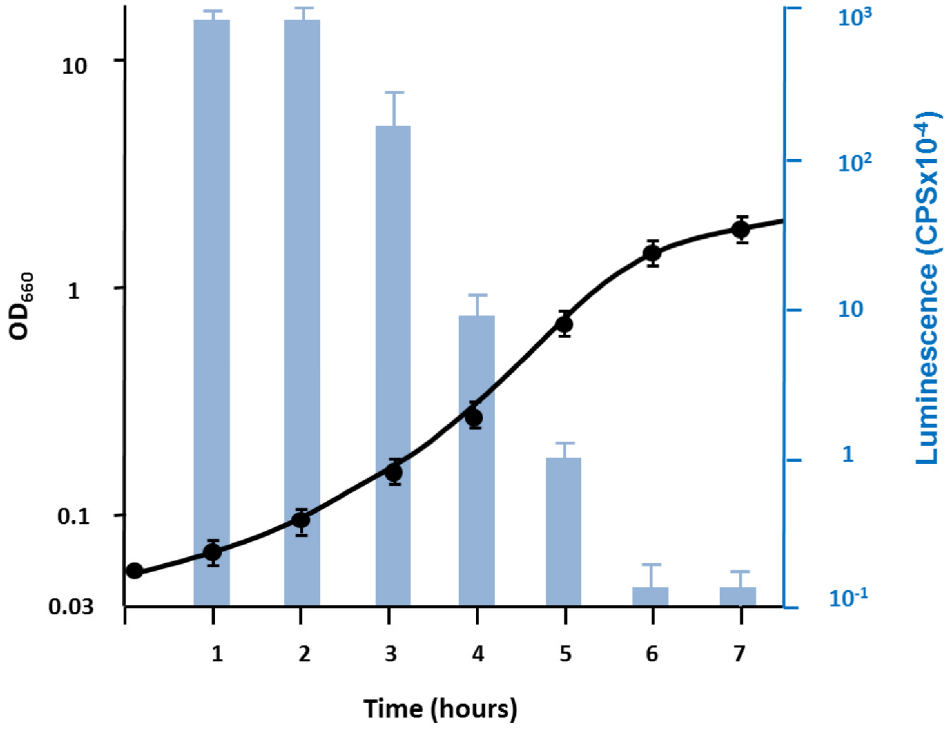

Figure S3. Accumulation of adenylate-cyclase (AC) activity in *B. anthracis* culture supernatant. The presence of EF in samples collected from a *B. anthracis* culture (in DMEM in 10% CO_2 atmosphere) was determined by the ATP-depletion assay at the indicated time-points. The luminescence-inhibition is indicated by the blue histograms (right axis in blue). The growth profile of the culture is indicated by the black curve (left axis in black) super-imposed on the luminescence-inhibition determined by the ATP-depletion assay.

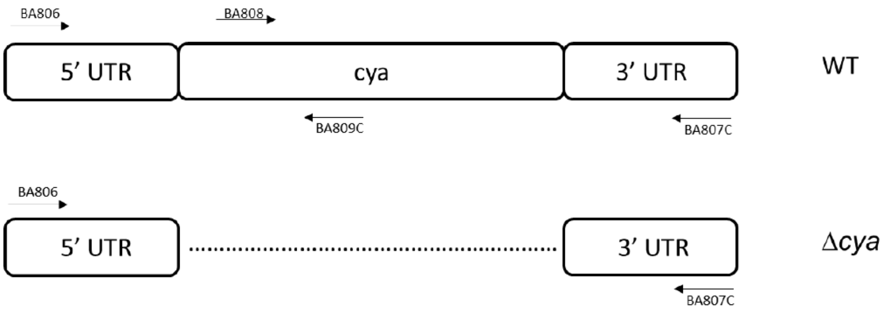

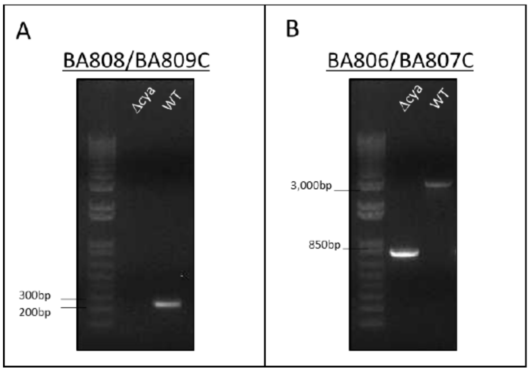

PCR Reaction	BA806	BA807C	BA808	BA809C	Δcya	WT
A	+	+	-	-	884	3194
B	-	-	+	+	---	252

BA806- TAACCTGCTGAATTCAAAATCGGACTTAG
BA807C- CCCTGGTTTAATCGCATCATTA
BA808- GAAGAGAAA AATATGATGAATAGTAGAGGTG
BA809C- GGTCGCTACTATCACTATCATGCG

Figure S4. Confirmation of the *cya*-gene disruption by Polymerase-chain reaction (PCR) analysis. PCR reactions were carried-out using as templates chromosomal DNA prepared from Colony No. 3 (which exhibited abrogation of the AC activity see Figure 3) and parental wild-type (WT) strain. The deletion of the *cya* gene is schematically depicted in the upper panel as a dotted line. UTR (un-translated region) refers to the homology fragments flanking the *cya* locus which were used for the targeted disruption. The position of the primers used for the PCR reactions is indicated as arrows. The expected length (in base pairs) of the PCR products as well as the DNA-sequence of the primers are indicated in the table in the right-lower panel. The PCR products were further analyzed by DNA sequencing which confirmed the deletion of the *cya* gene. The presence (+) or absence (-) of a PCR product are indicated in the table in the lower-right panel.
